# Complement Activation *via* the Lectin and Alternative Pathway in Patients With Severe COVID-19

**DOI:** 10.3389/fimmu.2022.835156

**Published:** 2022-02-02

**Authors:** Janina Niederreiter, Christine Eck, Tajana Ries, Arndt Hartmann, Bruno Märkl, Maike Büttner-Herold, Kerstin Amann, Christoph Daniel

**Affiliations:** ^1^ Department of Nephropathology, University Hospital Erlangen, Friedrich-Alexander-University (FAU) Erlangen-Nürnberg, Erlangen, Germany; ^2^ Institute of Pathology, University Hospital Erlangen, Friedrich-Alexander-University (FAU) Erlangen-Nürnberg, Erlangen, Germany; ^3^ General Pathology and Molecular Diagnostics, Medical Faculty Augsburg, University Augsburg, Augsburg, Germany

**Keywords:** complement deposition, COVID-19, kidney, lung, autopsy, lectin pathway

## Abstract

Complement plays an important role in the direct defense to pathogens, but can also activate immune cells and the release of pro-inflammatory cytokines. However, in critically ill patients with COVID-19 the immune system is inadequately activated leading to severe acute respiratory syndrome (SARS) and acute kidney injury, which is associated with higher mortality. Therefore, we characterized local complement deposition as a sign of activation in both lungs and kidneys from patients with severe COVID-19. Using immunohistochemistry we investigated deposition of complement factors C1q, MASP-2, factor D (CFD), C3c, C3d and C5b-9 as well as myeloperoxidase (MPO) positive neutrophils and SARS-CoV-2 virus particles in lungs and kidneys from 38 patients who died from COVID-19. In addition, tissue damage was analyzed using semi-quantitative scores followed by correlation with complement deposition. Autopsy material from non-COVID patients who died from cardiovascular causes, cerebral hemorrhage and pulmonary embolism served as control (n=8). Lung injury in samples from COVID-19 patients was significantly more pronounced compared to controls with formation of hyaline membranes, thrombi and edema. In addition, in the kidney tubular injury was higher in these patients and correlated with lung injury (r=0.361*). In autopsy samples SARS-CoV-2 spike protein was detected in 22% of the lungs of COVID-19 patients but was lacking in kidneys. Complement activation was significantly stronger in lung samples from patients with COVID-19 *via* the lectin and alternative pathway as indicated by deposition of MASP-2, CFD, C3d and C5b9. Deposits in the lung were predominantly detected along the alveolar septa, the hyaline membranes and in the alveolar lumina. In the kidney, complement was significantly more deposited in patients with COVID-19 in peritubular capillaries and tubular basement membranes. Renal COVID-19-induced complement activation occurred *via* the lectin pathway, while activation of the alternative pathway was similar in both groups. Furthermore, MPO-positive neutrophils were found in significantly higher numbers in lungs and kidneys of COVID-19 patients and correlated with local MASP-2 deposition. In conclusion, in patients who died from SARS-CoV-2 infection complement was activated in both lungs and kidneys indicating that complement might be involved in systemic worsening of the inflammatory response. Complement inhibition might thus be a promising treatment option to prevent deregulated activation and subsequent collateral tissue injury in COVID-19.

## Introduction

Since December 2019 the new coronavirus SARS-CoV-2 has spread around the world creating a worldwide pandemic. Despite vaccination campaigns in progress, we continue to see severe cases of COVID-19 with patients requiring intensive care treatment with frequent fatal outcome. The lungs are the primary manifestation of COVID-19 with patients experiencing fever, cough and dyspnea and in severe cases acute respiratory distress syndrome (ARDS) (1, 2). Histopathological alterations include diffuse alveolar damage, necrosis of pneumocytes, inflammatory infiltrate and formation of hyaline membranes (3). Due to the ability of SARS-CoV-2 to bind to the ACE2 receptor expressed by a large range of cells multiple other organs have been reported to be affected by COVID-19 (4, 5). Particularly, the kidney is involved in many patients with a considerable proportion of hospitalized patients experiencing primarily acute kidney injury (AKI). In addition, the occurrence of AKI in severe COVID-19 is associated with a higher mortality (6, 7). Some studies suggest direct viral infection of glomerular and tubular cells through the ACE2-receptor possibly causing acute proximal tubular injury and detachment of podocytes (7–10). However, other findings implicate indirect pathomechanisms being responsible for kidney injury including hemodynamic effects through sepsis-related factors, microangiopathy, cytokine storm and excessive immune response causing inflammation and triggering tissue damage (11–13). One pathway potentially involved in the SARS-CoV-2 driven inflammatory cytokine overproduction is activation of the complement system (12). This system, which belongs to the innate immune system, acts as a crucial component in the defense against infection by opsonizing pathogens or damaged cells, attracting and activating leukocytes or directly lysing bacteria or cells through the membrane attack complex. Initiation of the complement cascade can be done by three different pathways, the classical pathway, the lectin pathway, and the alternative pathway (14, 15). The classical pathway is activated by immune complexes and many other self and non-self molecules binding to C1q, leading to a conformational change, and activating serine proteases C1s and C1r (16). To activate the lectin pathway plasma-circulating lectins (collectins like mannan-binding lectin and ficolins) recognize carbohydrate patterns on the surface of microorganisms, called pathogen-associated molecular patterns (PAMPs). By binding dimers of mannan-binding lectin-associated serine protease 2 (MASP-2) these pattern-recognition molecules (PRM) form a complex which leads to the cleavage of C4 and C2 (17, 18). In contrast, the alternative pathway is constantly active at a low level and is initiated by spontaneous hydrolysis of C3. Hydrolysed C3 binds to factor B (CFB) which acts as a substrate to serine protease factor D (CFD), resulting in formation of a C3 convertase (15, 19). In the end, all 3 complement pathways can lead to C3 convertase activation, followed by activation of C5 convertase triggering the formation of the membrane attack complex (MAC) by C5b-9. MAC consists of different complement factors, binds to the cells and causes cell lysis by producing pores in the plasma membrane (15). Cleavage products C3a and C5a are potent inflammatory peptides capable of inducing the release of cytokines, thus contributing to the development of a cytokine storm, and recruitment of inflammatory cells (12, 15, 20). Meta-analysis of studies investigating systemic complement activation using serum samples of patients with COVID-19 demonstrated that lower C3 and C4 serum levels were significantly associated with higher COVID-19 severity and mortality (21). However, evidence of COVID-19-induced complement activation in organs has only been provided in a few studies using small sample numbers to date (22–25). In an earlier study, we could demonstrate complement activation in renal samples from patients with COVID-19 (24). However, this study was limited by a small sample size, the wide range in severity of COVID-19 disease, and the inclusion of patients with various renal diseases. In this study, we extended the evaluation of complement activation in the kidney to the lung as the organ of primary injury, included more patients and focused on severe COVID-19 cases by using autopsy material from patients who died of COVID-19. After exclusion of samples with severe tissue autolysis, 38 patients have been included for analysis of complement deposition in various compartments in lungs and kidneys. A group of 8 postmortem organ samples was selected as control.

## Materials and Methods

### Human Renal Tissue Specimens

To evaluate the relevance of complement in mediating severe lung and renal pathology during COVID-19 infection tissue was collected in a standardized manner during autopsy from patients who died due to SARS-CoV-2 infection (26, 27). The diagnosis of COVID-19 was confirmed by RT-PCR analysis. We included two cohorts, one from Pathology Institutes University Medical Center Augsburg (n=36) and a second from University Erlangen-Nürnberg (n=15). In a first step, we pre-evaluated the quality of all available autopsy material and excluded all samples with severe autolysis. Finally, we included 25 samples from the Augsburg collective and 13 samples from the Erlangen cohort. Autopsy material of patients who died from cerebral hemorrhage (n=2), pulmonary embolism (n=2) and cardiovascular disease (n=4) collected from the archive of the Department of Pathology at FAU served as controls. Analysis was approved by the local Ethics committees (Ethics committee of the Friedrich-Alexander University (FAU) Erlangen-Nürnberg, reference number 4415 and internal review board of the Medical Center-Augsburg (BKF No. 2020–18) and the ethics committee of the University of Munich (Project number 20–426, COVID-19 registry of the University Hospital Augsburg). Patients’ characteristics are described in **Table 1** and controls in **Table 2**.

**Table 1:** Clinical findings in patients with COVID-19 infection.

Pt	Age	Sex	Duration of illness [days]	Comorbidities							Acute kidney injury
				Cardiovascular	Hypertension	Cancer	Diabetes	Obesity	Chronic respiratory disease	Chronic kidney disease	
1	64	M	25	n	n	y	n	n	n	n	y
2	83	M	8	y	n	n	n	n	n	n	n
3	85	M	6	y	y	n	n	n	n	n	n
4	90	F	1	y	y	n	n	n	n	n	y
5	85	M	8	n	y	y	n	n	n	y	n
6	69	M	5	y	y	n	y	y	n	y	y
7	83	M	10	y	n	n	y	y	n	n	y
8	60	M	30	n	n	n	n	n	n	n	y
9	61	F	22	n	y	n	n	y	n	n	y
10	89	M	36	y	y	n	n	n	n	y	n
11	64	M	9	y	y	n	y	y	y	n	y
12	83	M	18	y	y	n	n	n	n	n	y
13	87	M	6	y	y	n	n	n	y	n	n
14	51	F	6	y	n	n	n	y	n	y	n
15	66	M	7	y	y	n	n	n	n	y	y
16	65	M	30	n	n	y	n	n	n	n	y
17	62	M	44	n	n	n	n	n	n	n	y
18	70	M	6	y	n	n	y	n	n	y	y
19	85	M	2	y	y	n	n	n	n	y	y
20	79	F	13	n	n	n	n	n	n	n	n
21	77	M	18	y	y	n	y	n	y	n	y
22	74	M	3	y	y	n	y	y	n	y	n
23	90	F	14	y	y	y	n	n	y	n	n
24	92	F	16	y	n	y	n	n	y	n	n
25	73	M	64	y	y	y	n	n	y	n	y
26	76	F	8	n	y	n	n	n	n	y	y
27	92	F	17	n	n	n	n	n	n	y	y
28	92	F	5	y	n	n	n	n	n	n	n
29	83	F	unknown	y	y	y	n	n	n	n	y
30	59	M	60	y	n	n	n	n	n	n	n
31	89	F	unknown	y	y	n	y	n	n	n	y
32	82	M	4	y	y	n	n	n	n	n	unknown
33	82	F	23	y	y	n	n	n	y	n	n
34	74	M	38	y	y	y	y	n	n	n	n
35	70	M	6	y	n	y	n	n	n	n	y
36	77	M	8	y	y	n	n	n	n	n	unknown
37	74	M	3	y	n	y	n	n	y	n	y
38	58	M	39	y	y	n	n	n	n	n	y
	**76.2 ± 11.3**	**26:12**	**17.2 ± 16.1**	**29/38**	**23/38**	**10/38**	**8/38**	**6/38**	**8/38**	**10/38**	**22/38**

Pt, patient; M, male; F, female; blue shading = patients from the Augsburg collective; no shading = patients from the Erlangen collective. Bold values represent mean+/-SD or ratios.

**Table 2:** Cause of death in control patients.

Pt	Age	Sex	Cause of death
1	56	M	Congestive heart failure with cerebral hemorrhage
2	71	F	Hemodynamic shock with acute bowel ischemia
3	49	M	Heart failure with gastric cancer
4	61	F	Pulmonary embolism
5	59	M	Cerebral compression with cerebral hemorrhage
6	48	F	Cardiac arrhythmia
7	57	F	Acute myocardial infarction with aortic valve replacement
8	75	F	Pulmonary embolism
	**59.5 ± 9.5**	**3:5**	

Pt, patient; M, male; F, female. Bold values represent mean+/-SD or ratios.

### Immunohistochemistry

For immunohistochemical stainings formalin-fixed paraffin-embedded (FFPE) lung and kidney samples were cut into 2 µm sections, deparaffinized and rehydrated. Antigen retrieval was performed using pronase E (Sigma Aldrich, Taufkirchen, Germany) digestion for 30-45 minutes at 37°C (C1q, C3c, C5b-9) or cooking in target retrieval solution pH 6 (DAKO Deutschland GmbH, Hamburg, Germany) for 2.5 minutes (C3d, C4d, CFD, MASP-2, COVID-19 spike protein, MPO). Endogenous peroxidase was blocked with 3% H_2_O_2_ and unspecific antigens with Avidin-Biotin (Vector laboratories, Burlingame, CA, USA) and normal goat or horse serum in blotto (1:5). The following primary antibodies were diluted in 50 mM Tris pH 7.4 and incubated over-night at 4°C: C1q, a rabbit polyclonal antibody against human C1q (A0136; DAKO Deutschland GmbH); C3c, a rabbit polyclonal antibody against human C3c (A0062; DAKO Deutschland GmbH); C3d, a rabbit monoclonal antibody against human C3d (ab136916; Abcam, Cambridge, UK); C4d, a rabbit polyclonal antibody against human C4d (RBK039-05; Zytomed Systems GmbH, Berlin, Germany); C5b-9, a mouse monoclonal antibody against human C5b-9 (M0777; DAKO Deutschland GmbH); complement factor D (CFD), a rabbit polyclonal antibody against rat cross-reactive to human CFD (PA5-79035; Thermo Fisher Scientific, Carlsbad, CA, USA); MASP-2, a rabbit polyclonal antibody against human Mannan-binding lectin serine peptidase 2 [HPA029313; Sigma Aldrich; specificity of the antibody was supported by positive staining in liver (**Supplementary Figure 1**)]; MPO a rabbit polyclonal antibody against human myeloperoxidase (ab9535; Abcam, Cambridge, UK), and SARS-CoV-2 spike protein, a mouse monoclonal antibody against SARS-CoV-2 spike protein (clone 224.2, kindly provided by M.-H. Jäck, Department of Molecular Immunology, FAU Erlangen-Nürnberg). After washing with 50 mM Tris pH 7.4, sections were incubated with biotinylated secondary goat anti-rabbit IgG (BA-1000; Vector laboratories) or horse anti-mouse IgG (BA-2001, Vector laboratories). Detection of bound antibodies was conducted using ABC-Kit and DAB-Impact as a substrate (both from Vector laboratories), while nuclei were counter stained with hemalaun. For negative controls primary antibody was substituted by antibody dilution buffer (50 mM Tris pH 7.4).

### Semi-Quantitative Evaluation of Complement in Lung and Renal Samples

Immunohistochemical staining in pulmonary and renal tissue was graded in different vascular and non-vascular compartments of the lung (i.e. alveolar septa, alveolar lumen, alveolar infiltrate, vessels and bronchioli) and the kidney (i.e. in glomeruli, peritubular capillaries, arteries, tubular basement membrane) using a semi-quantitative score (score 0-2 and 0-3, respectively), which described the distribution and intensity of the staining signal. For MPO staining, score 0 was defined as no positive neutrophilic in the respective compartment, score 1 was defined as single or few scattered positive neutrophil cells in a compartment and score 2 was defined as the clusters of positive neutrophils. For the components of the complement system, score 0 was chosen for samples showing no specific reactivity in the respective compartment, score 1 represented samples showing low reactivity in single small patches. Score 2 was defined as reactivity in larger patches scattered throughout the sample and score 3 was defined as reactivity in large patches making up the majority of the sample. For compartments showing pronounced staining reactivity [i.e. peritubular capillaries and tubular basement membrane (TBM)] multiple fields of view were graded and mean values representing the distribution of reactivity in a sample were formed. In order to display the total staining of a sample, sum scores that included all individual scores for each compartment were generated.

### Injury Scores

An additional qualitative score was used to assess lung injury, which included common pathological changes in COVID-19 such as edema, thrombi, inflammation, hyaline membranes and necrosis. The occurrence of each of these changes was assigned a point score, resulting in a maximum score of 5. Renal damage was evaluated using semi-quantitative scores to assess glomerulosclerosis (GSI, including glomerular matrix accumulation) and tubulointerstitial injury (TSI, including tubular interstitial fibrosis and inflammation) as described previously (28).

### Statistical Analyses

After testing for normal distribution of values using Kolmogorov-Smirnov test, data were analyzed using Mann-Whitney test. Results of the evaluated immunohistochemistry were correlated with clinical data using Spearman test. In all tests p<0.05 was accepted as statistically significant. Statistical analyses were performed using GraphPad Prism 8 for Windows software (version 8.3, GraphPad software Inc., San Diego, CA, USA) and SPSS Statistics 28 (IBM, Armonk, NY, USA).

## Results

### Patients With Severe COVID-19 Showed Lung and Kidney Damage

**Figure 1:**
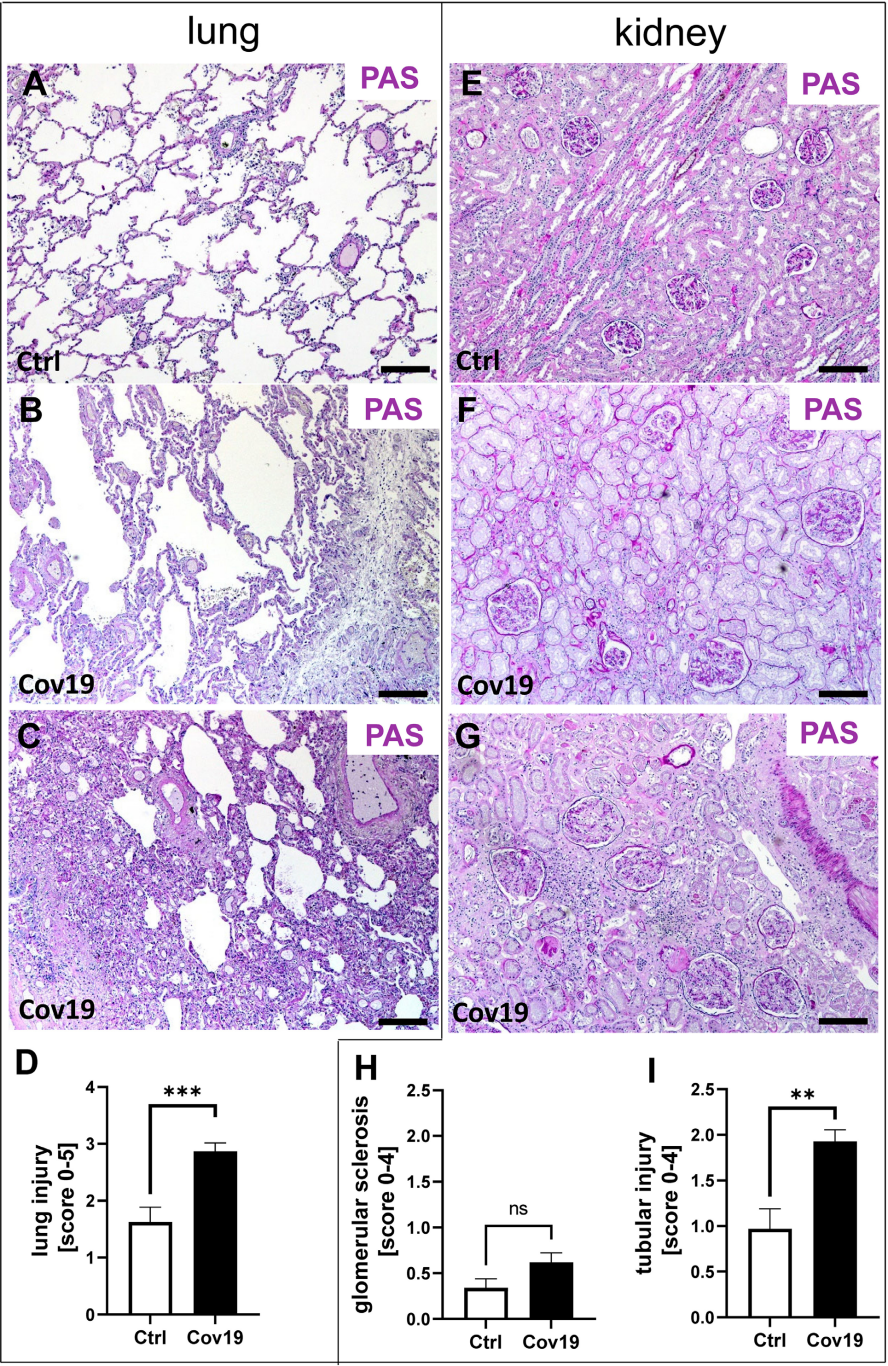
Pulmonary and renal injury in patients with COVID-19. Representative pictures of PAS-stained lung and kidney specimens from a control patient **(A, E)** and patients with COVID-19 **(B, C, F, G)**. Lungs of a control patient showing mild changes with little alveolar accumulation of inflammatory cells **(A)**, while changes in lung samples from patients with COVID-19 ranged from mild pulmonary injury including accumulation of alveolar inflammatory cells and interstitial edema **(B)** to severe pulmonary injury with collapsed alveoli, dense alveolar infiltrate, and congested capillaries **(C)**. The extent of lung injury in control patients (Ctrl, n = 8) versus COVID-19 patients (Cov19, n = 38) was analyzed using PAS-stained lung samples and semi-quantitative scoring **(D)**. While kidneys in controls showed only minor pathological changes **(E)**, kidney samples from COVID-19 patients exhibited mild **(F)** to more severe **(G)** kidney injury. Renal injury was determined using semi-quantitative scoring of glomerular sclerosis **(H)** and acute and chronic tubular injury **(I)**. ns, non-significant; Scale bar = 200μm; **p < 0.01; ***p < 0.001.

**Figure 2:**
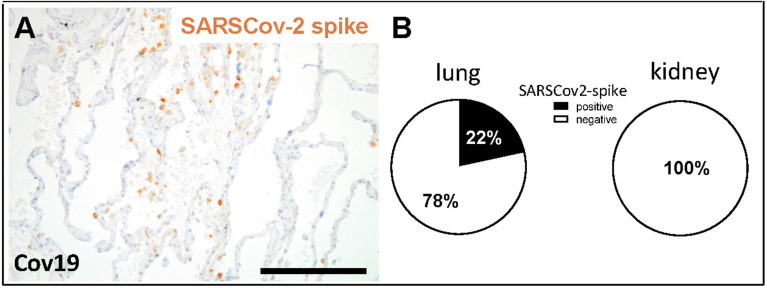
Detection of SARS-CoV-2 spike protein in lung and kidney samples. SARS-CoV-2 spike protein was detected in lung samples from patients with COVID-19, as shown in a representative micrograph of immunohistochemistry (**A**, brown staining). Lung and kidney samples from COVID-19 patients were analyzed for positive staining for SARSCov-2 spike protein and the percentage of positive samples are shown **(B)**. Scale bar = 200μm.

In total 38 patients who died of COVID-19 with a proportion of 26 males and 12 females with a mean age of 76.2± 11.3 years were included in the study (**Table 1**). The average time between first diagnosis and death was 17.2 ± 16.1 days. More than half of the Covid-19 patients had cardiovascular changes and hypertension as comorbidities (**Table 1**). Further comorbidities were cancer (10/38), diabetes (8/38), obesity (6/38), chronic respiratory disease (8/38) and chronic kidney disease (10/38) (**Table 1**). In 22 of 38 patients acute kidney injury was observed during COVID-19 disease (**Table 1**). In the control group we included eight patients, three males and five females with an average age of 59.5 ± 9.5 years who died of cardiac diseases including heart failure, cardiac arrhythmia or myocardial infarction or pulmonary embolism or hemodynamic shock with acute bowel ischemia (**Table 2**). Lungs from control patients showed mild changes with little alveolar infiltrate (**Figure 1A**), while changes in lungs of patients with COVID-19 showed signs of different stages of diffuse alveolar damage (DAD) with hyaline membranes, thickening af the alveolar septa and interstitial fibroblastic proliferation (**Figure 1B**) as well as mononuclear inflammatory infiltrates (**Figure 1C**). Lung injury in patients with COVID-19 was significantly stronger compared to controls, as assessed by qualitative scoring (**Figure 1D**). Most kidney sections of controls showed at least mild changes in glomeruli and within the tubulointerstitium (**Figure 1E**). In contrast, changes of kidney tissue from COVID-19 patients ranged mild (**Figure 1F**) to severe (**Figure 1G**) including tubular injury, tubular necrosis, mild glomerular sclerosis, interstitial infiltrates, and interstitial fibrosis. However, while glomerular injury was comparably low in both groups (**Figure 1H**), tubular injury was significantly higher in samples from COVID-19 patients (**Figure 1I**). Interestingly, kidney injury positively correlated with lung injury score (r=0.364*, p=0.013). SARS-CoV-2 virus could be detected in 22% of all investigated lungs and none of the kidney samples from COVID-19 patients (**Figures 2A, B**).

### Deposition of Classical Complement Factor C1q Was Weak in Lungs and Kidneys of Patients With COVID-19

**Figure 3:**
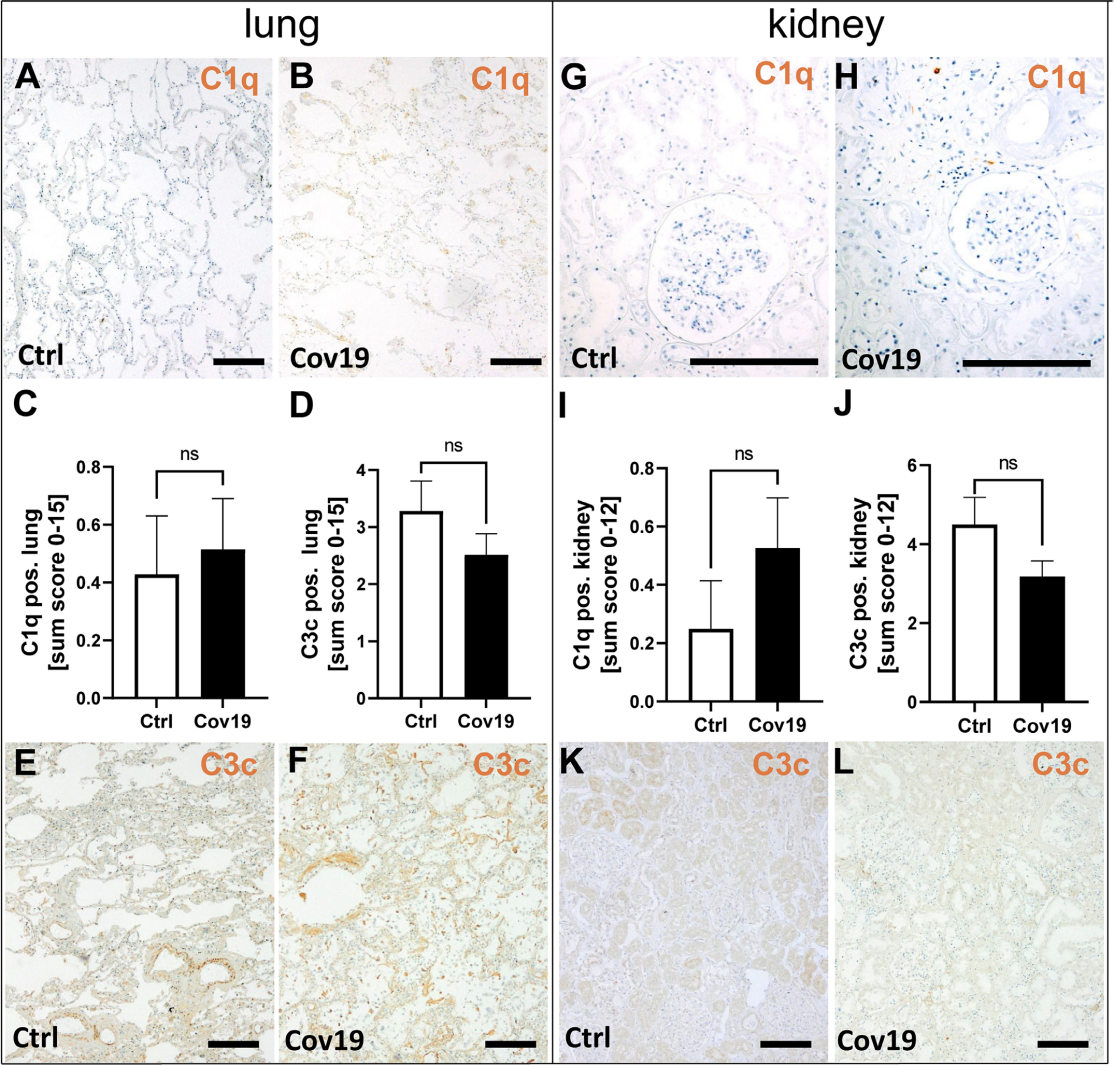
Detection of the classical pathway component C1q and the stable complement cleavage product C3c in samples from patients with COVID-19. Representative microgaphs of immunohistochemical staining for C1q in lung **(A, B)** and kidney **(G, H)** samples of a control patients **(A, G)** and a with COVID-19 patient **(B, H)** are shown. Sum scores of semi-quantitative evaluation of C1q **(C)** and C3c **(D)** deposition in lung demonstrate weak staining in both investigated groups. Representative pictures of immunohistochemical staining for C3c in lung **(E, F)** and kidney **(K, L)** samples of control patients **(E, K)** and patients with COVID-19 **(F, L)** are shown. In the kidney sum scores for semi-quantitative evaluation for C1q **(I)** and C3c **(J)** were low. ns, non-significant. Scale bar = 200 µm.

First, we investigated whether C1q, which is involved in activation of the classical pathway, was detectable in the lungs and kidneys of patients with COVID-19. C1q was barely detectable in the lungs and kidneys of controls (**Figure 3A**), but was also absent in both lungs (**Figures 3B, C**) and kidneys (**Figures 3G–I**) of COVID-19 patients which was reflected by sum scores below 1 (**Figures 3C, I**). Similarly, the detection of C3c, a stable cleavage product of C3, was detected only slightly more intensely than C1q in lung (**Figures 3D–F**) and kidney (**Figures 3J–L**). Here too, compared to the control group, the deposition of C3c was similar in COVID-19 patients in both lung (**Figure 3D**) and kidney (**Figure 3J**).

### Lectin Pathway Activator Mannan-Binding Lectin-Associated Serine Protease 2 (MASP-2) Was Markedly Deposited in Lungs and Kidneys From Patients With COVID-19

**Figure 4:**
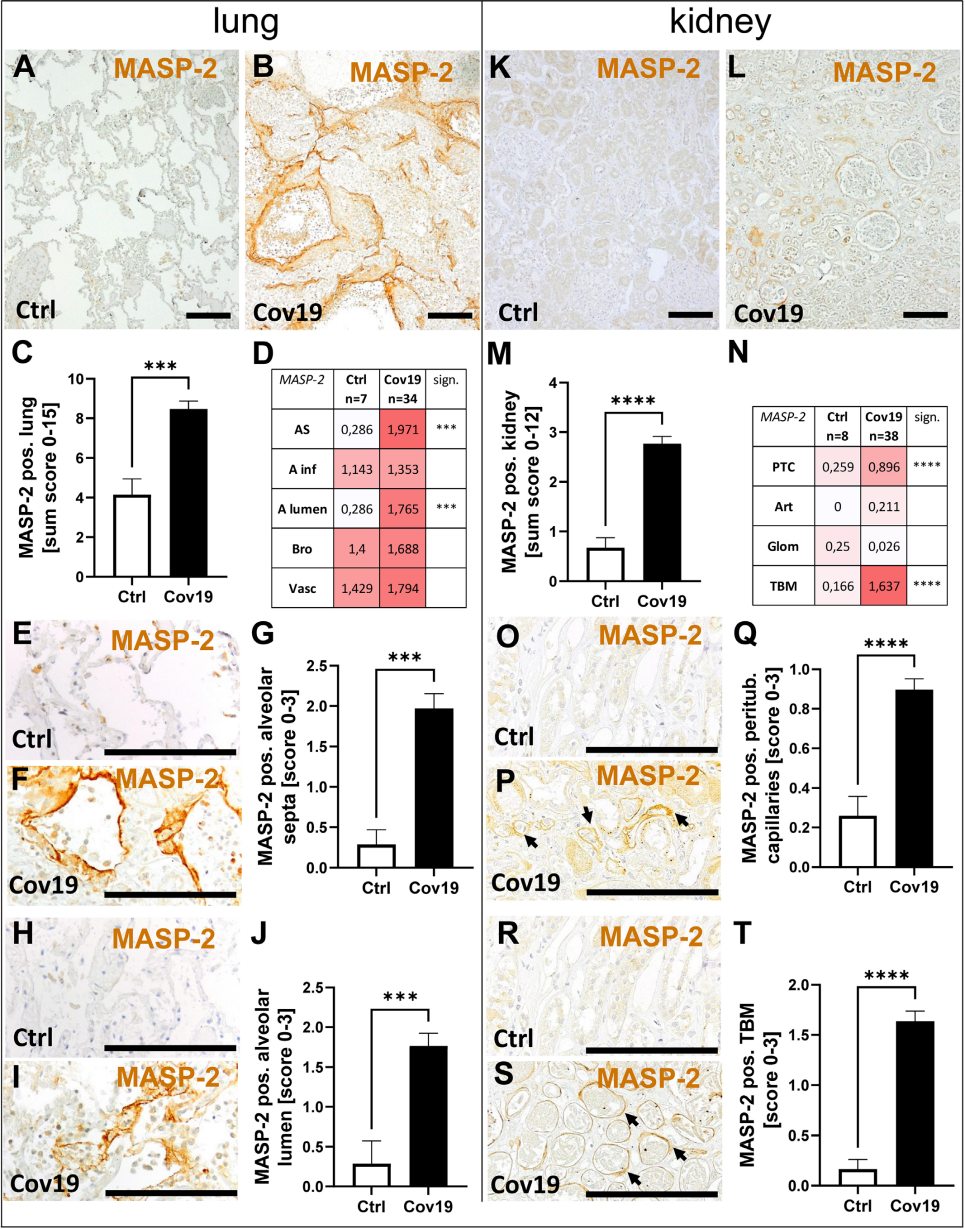
Detection of the lectin pathway component MASP-2 in lung and kidney samples from patients with COVID-19. Overview of immunohistochemical staining for mannose-binding protein-associated serine protease 2 (MASP-2) in lung samples of a control patient **(A)** and a patient with COVID-19 **(B)** was shown. Results of semi-quantitative scoring in multiple compartments for MASP-2 deposition in the lung were summarized in a sum score **(C)** and overview of the mean values of compartment-specific analyses is given in a heat map **(D)**. Representative MASP-2 stainings in alveolar septa of a control patient **(E)** and a COVID-19 patient **(F)** and comparative analyses are shown **(G)**. MASP-2 staining in alveolar lumina in a control patient **(H)** and a patient with COVID-19 **(I)** and statistical comparison of both groups **(J)**. Overview of MASP-2 staining in kidney samples of a control patient **(K)** and a patient with COVID-19 **(L)**. Results of semi-quantitative scoring in multiple compartments for MASP-2 deposition in the kidney are summarized in a sum score **(M)** and overview of the mean values of compartment-specific analyses is given in a heat map **(N)**. Representative MASP-2 staining in peritubular capillaries of a control patient **(O)** and a COVID-19 patient (**P**, arrows) and a comparative analyses **(Q)**. MASP-2 staining of tubular basement membrane in a control patient **(R)** and a patient with COVID-19 (**S**, arrows) and comparison of both groups **(T)**. AS, alveolar septa; A inf, accumulation of alveolar inflammatory cells; A lumen, alveolar lumen; Bro, bronchioles; Vasc, vascular; sign., significance; PTC, peritubular capillaries; Art, arterial; Glom, glomerular; TBM, tubular basement membrane; BM, basement membrane. Scale bar = 200µm; ***p < 0.001; ****p < 0.0001.

In contrast, MASP-2 as an activator of the lectin pathway was significantly more abundant in the lungs of patients with COVID-19 compared to controls (**Figures 4A–C**). Compartment-specific analysis showed that MASP-2 was detectable in the bronchioli, the larger vessels, and within alveolar infiltrates in controls and COVID-19 cases in comparable quantities (**Figure 4D**). In contrast, in the alveolar septa (AS) and in the lumina of the alveoli (A lumen) it was almost exclusively present in the samples from patients with COVID-19 (**Figures 4D–J**). In particular, hyaline membranes in the lungs of patients with COVID-19 showed strong MASP-2 deposition (**Figure 4F**). We detected smaller amounts of MASP-2 in the kidneys compared to the lungs (**Figures 4K, L**). However, the MASP-2 sum score for the kidneys of patients with COVID-19 was on average more than 3-fold higher (**Figure 4M**). MASP-2 deposition in the kidneys of COVID-19 patients was seen in peritubular capillaries (PTC) and especially in tubular basement membranes (TBM), with almost 10 times higher levels than in the control group (**Figures 4N–T**). In contrast, we could hardly find MASP-2 in the renal arteries (Art) and glomeruli (Glom) (**Figure 4N**).

### Alternative Pathway Activator Factor D (CFD) Was Markedly Increased in Lungs From Patients With COVID-19, But Not in Kidneys

**Figure 5:**
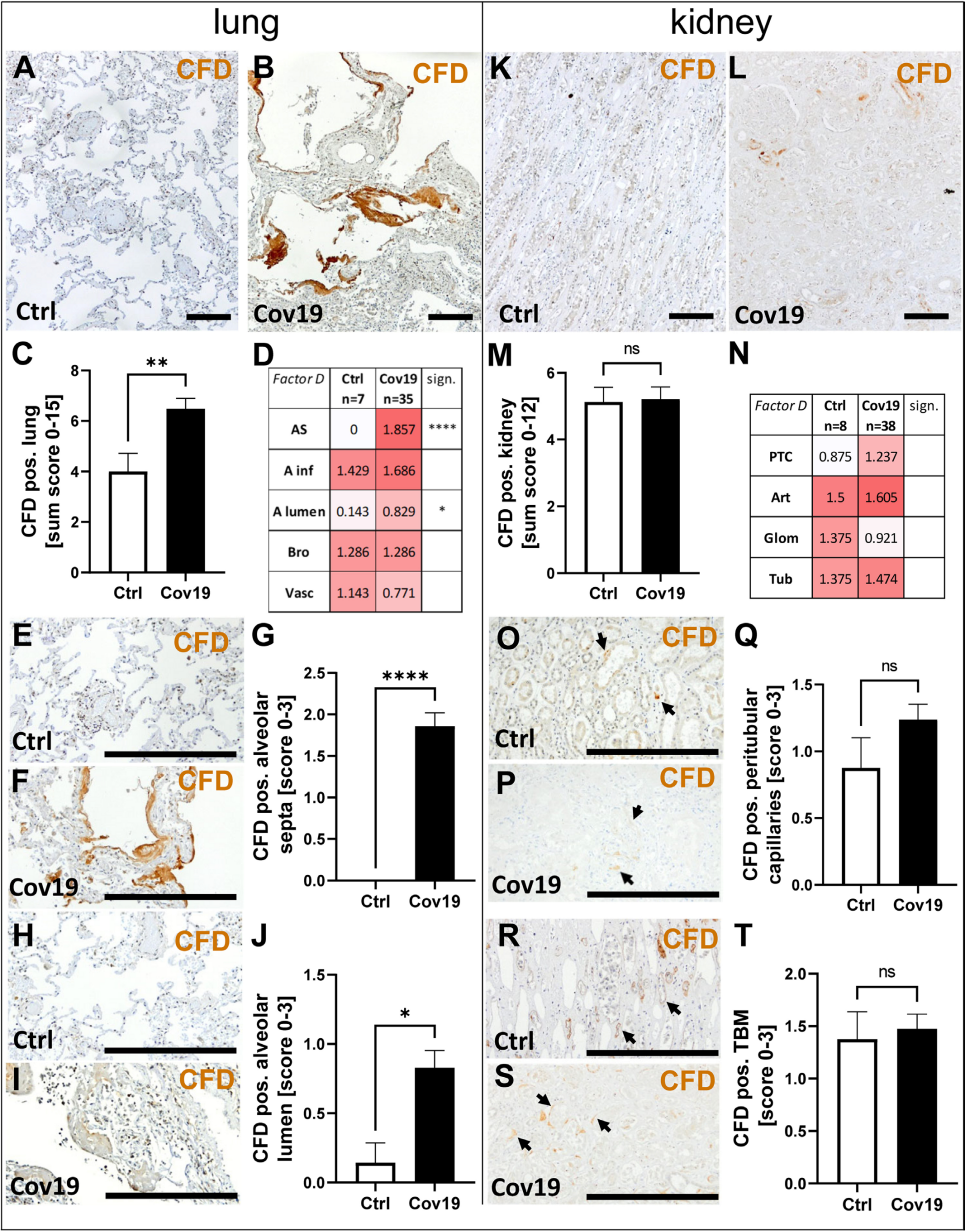
Detection of the alternative pathway component CFD in lung and kidney samples from patients with COVID-19. Overview of immunohistochemical staining for complement factor D (CFD) in lung samples of a control patient **(A)** and a patient with COVID-19 **(B)** was shown. Results of semi-quantitative scoring in multiple compartments for CFD deposition in the lung were summarized in a sum score **(C)** and overview of the mean values of compartment-specific analyses is given in a heat map **(D)**. Representative CFD stainings in alveolar septa of a control patient **(E)** and a COVID-19 patient **(F)** and comparative analyses are shown **(G)**. CFD staining in alveolar lumina in a control patient **(H)** and a patient with COVID-19 **(I)** and statistical comparison of both groups **(J)**. Overview of CFD staining in kidney samples of a control patient **(K)** and a patient with COVID-19 **(L)**. Results of semi-quantitative scoring in multiple compartments for CFD deposition in the kidney are summarized in a sum score **(M)** and overview of the mean values of compartment-specific analyses is given in a heat map **(N)**. Representative CFD staining in peritubular capillaries of a control patient **(O)** and a COVID-19 patient (**P**, arrows) and a comparative analyses **(Q)**. CFD staining of tubular basement membrane in a control patient **(R)** and a patient with COVID-19 (**S**, arrows) and comparison of both groups **(T)**. AS, alveolar septa; A inf, accumulation of alveolar inflammatory cells; A lumen, alveolar lumen; Bro, bronchioles; Vasc, vascular; sign., significance; ns, non-significant; PTC, peritubular capillaries; Art, arterial; Glom, glomerular; TBM, tubular basement membrane; BM, basement membrane. Scale bar = 200µm; *p < 0.05; **p < 0.01; ****p < 0.0001.

To investigate additional complement activation *via* the alternative pathway, we analyzed the activator factor D (CFD). Interestingly, this factor was also significantly more abundant in the lungs of patients with COVID-19 compared to controls (**Figures 5A–C**). Compartment-specific analysis showed that CFD was detectable in alveolar infiltrates, bronchioles and the larger vessels in controls and COVID-19 cases in comparable quantities (**Figure 5D**). Similar to the staining pattern of MASP-2, CFD was significantly more deposited in the area of the alveolar septa (**Figure 5D**), especially the hyaline membranes (**Figures 5E–G**), and in the lumina of the alveoli (**Figures 5H–J**). In contrast, the sum score for CFD in the kidneys of COVID-19 patients was comparable to that of controls (**Figures 5K–M**). Compartment-specific analysis for CFD showed slightly increased deposition in peritubular capillaries, but did not reach the significance level (**Figures 5N–Q**) and CFD-positive TBM were comparable in both groups (**Figures 5R–T**).

### C3d Deposition Occurs in Both Lungs and Kidneys of Patients With COVID-19

**Figure 6:**
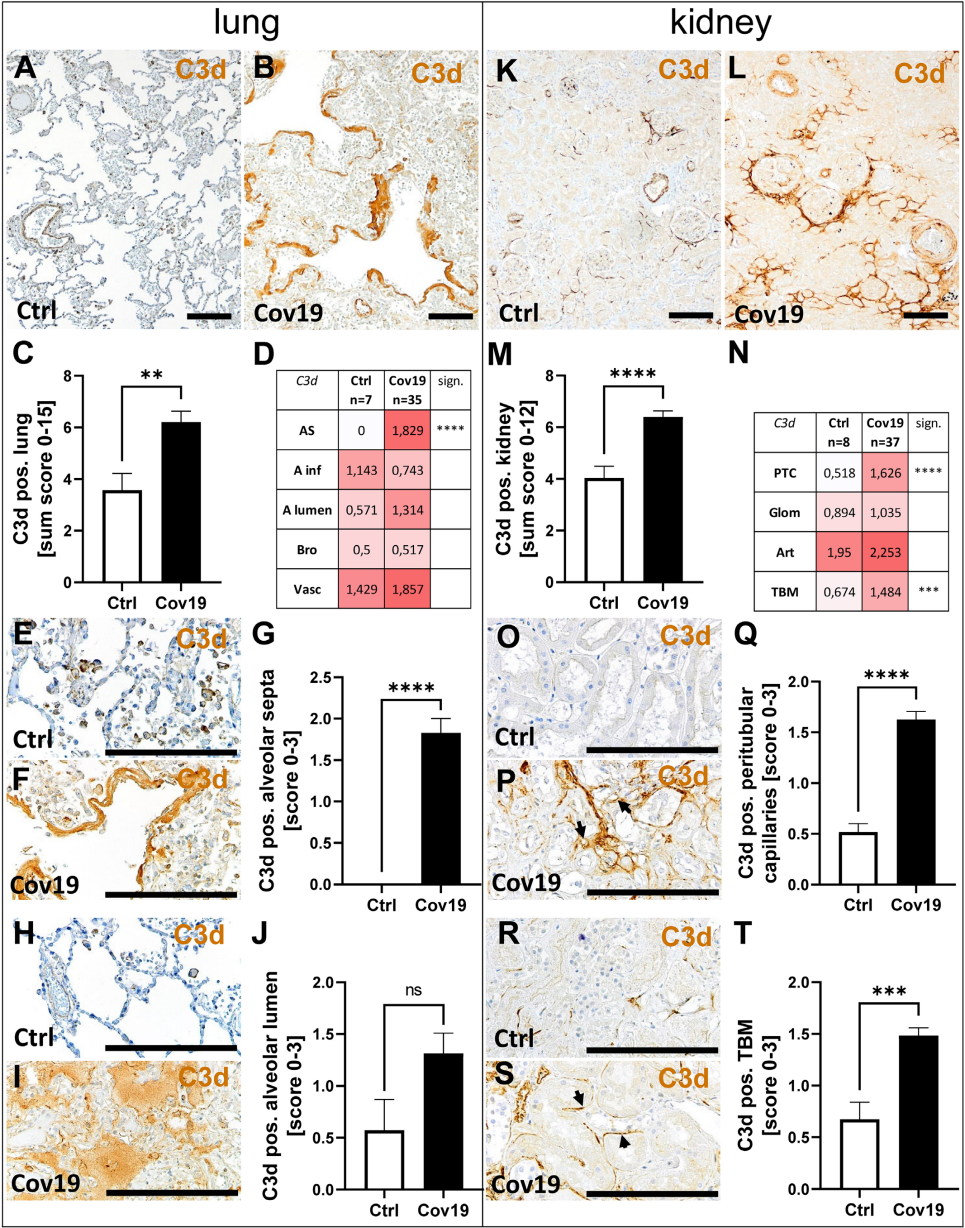
Detection of C3d in lung and kidney samples from patients with COVID-19. Overview of immunohistochemical staining for C3d in lungs of a control patient **(A)** and a patient with COVID-19 **(B)**. Results of semi-quantitative scoring in multiple compartments for C3d deposition in the lung summarized in a sum score **(C)** and overview of the mean values of compartment-specific analyses given in a heat map **(D)**. Representative C3d staining in alveolar septa of a control patient **(E)** and hyaline membranes in a COVID-19 patient **(F)** and statistical comparison of both groups **(G)**. C3d staining in alveolar lumina in a control patient **(H)** and a patient with COVID-19 **(I)** statistical comparison of both groups **(J)**. Overview of C3d staining in kidney biopsies of a control patient **(K)** and a patient with COVID-19 **(L)**. Results of semi-quantitative scoring in multiple compartments for C3d deposition in the kidney summarized in a sum score **(M)** and overview of the mean values of compartment-specific analyses given in a heat map **(N)**. Representative C3d staining in peritubular capillaries of a control patient **(O)** and a COVID-19 patient (**P**, arrows) and statistical comparison of both groups **(Q)**. C3d staining of tubular basement membrane in a control patient **(R)** and a patient with COVID-19 (**S**, arrows) and statistical comparison of both groups **(T)**. AS, alveolar septa; A inf, alveolar infiltrate; A lumen, alveolar lumen; Bro, bronchioles; Vasc, vascular; sign., significance; ns, non-significant; PTC, peritubular capillaries; Art, arterial; Glom, glomerular; TBM, tubular basement membrane; BM, basement membrane. Scale bar = 200µm; **p < 0.01; ***p < 0.001; ****p < 0.0001.

Next, we stained lung and kidney samples for C3d, the final degradation product of C3. In the lungs, as in MASP-2, significantly higher C3d amounts were observed looking at the sum scores in samples from patients with COVID-19 (**Figures 6A–C**). Striking, again, was the strong staining of the hyaline membranes for C3d which lie on top of the pneumocytes in the lungs of these patients (**Figure 6F**), which is absent in the controls (**Figures 6E, G**). In addition, C3d was found to a lesser extent in alveolar infiltrates and bronchioli (**Figure 6D**). The mean C3d deposition was more than doubled in the alveolae of COVID-19 patients, but did not reach the significance level compared with the control group (**Figures 6H–J**). Larger vessels also showed deposition of C3d, but this was comparable in both groups (**Figure 6D**). In the autopsy kidneys of both groups, C3d was well detectable and was significantly more prominent in the COVID-19 group compared with controls using the sum score, although the scores were only one third higher on average (**Figures 6K–M**). In the compartment-specific analysis, C3d deposition in the peritubular capillaries (**Figures 6N–Q**; PTC) and tubular basement membrane (**Figures 6N, R–T**; TBM) was significantly increased in the COVID-19 group, reaching levels approximately 3 times higher than the controls. In contrast, there was little difference in C3d deposition in glomeruli and arterial vessels (**Figure 6N**).

### The Correlation of MASP-2 and CFD With C3d and C5b-9 Deposition Suggests Complement Activation *via* the Lectin Pathway in Lungs and Kidneys and Additionally *via* the Alternative Pathway in Lungs of COVID-19 Patients

**Figure 7:**
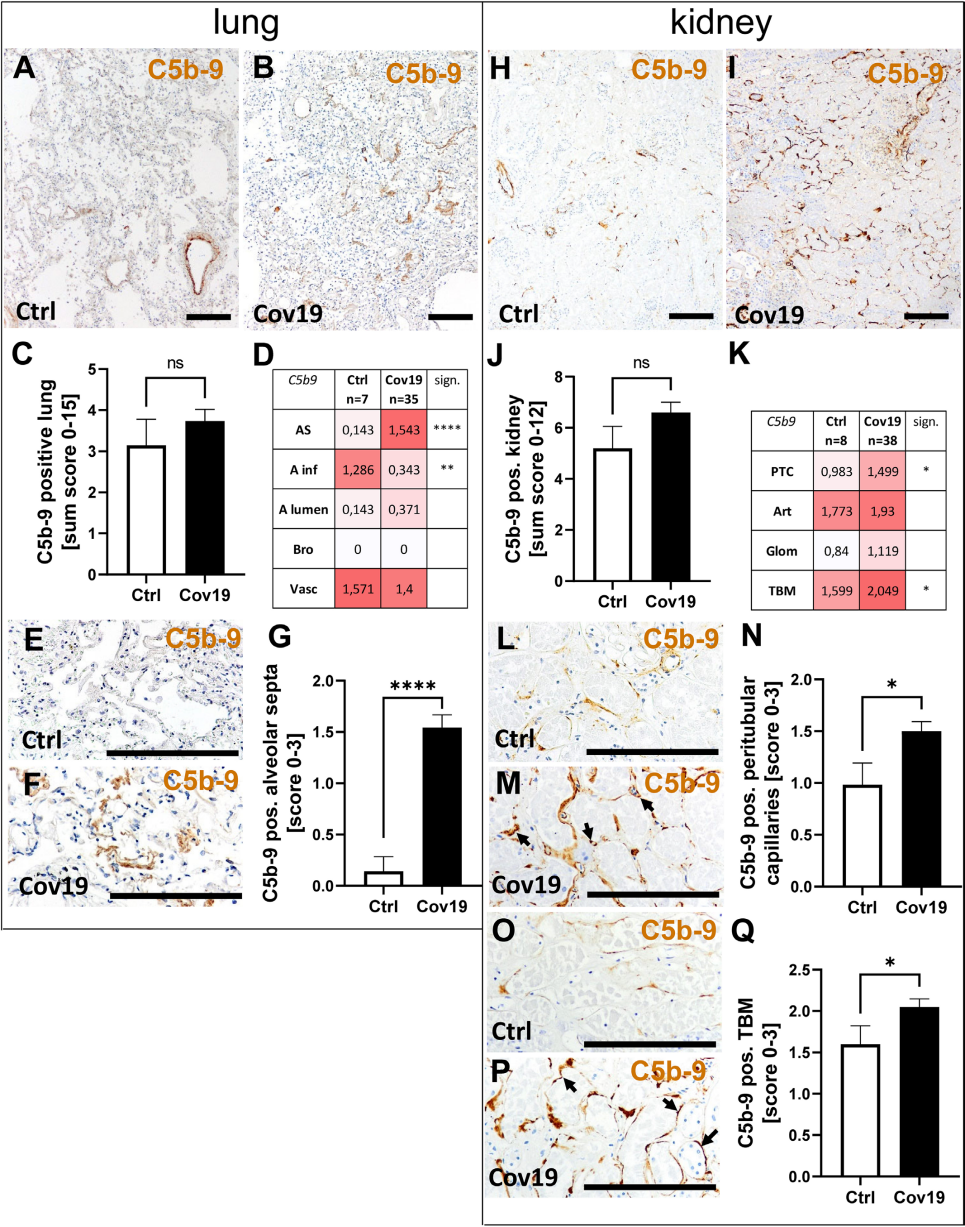
Detection of C5b-9 in lung and kidney specimens of COVID-19 patients. Overview of immunohistochemical stainings for C5b-9 in lungs of a control patient **(A)** and a patient with COVID-19 **(B)**. Results of semi-quantitative scoring in multiple compartments for C5b-9 deposition in the lung summarized in a sum score **(C)** and overview of the mean values of compartment-specific analyses given in a heat map **(D)**. Representative C5b-9 staining in alveolar septa of a control patient **(E)** and a COVID-19 patient **(F)** and statistical comparison of both groups **(G)**. Overview of C5b-9 staining in kidneysof a control patient **(H)** and a patient with COVID-19 **(I)**. Results of semi-quantitative scoring in multiple compartments for C5b-9 deposition in the kidney summarized in a sum score **(J)** and overview of the mean values of compartment-specific analyses given in a heat map **(K)**. Representative C5b-9 staining in peritubular capillaries of a control patient **(L)** and a COVID-19 patient (M, arrows) and statistical comparison of both groups **(N)**. C5b-9 staining of tubular basement membrane in a control patient **(O)** and a patient with COVID-19 (P, arrows) and statistical comparison of both groups **(Q)**. AS, alveolar septa; A inf, alveolar infiltrate; A lumen, alveolar lumen; Bro, bronchioles; Vasc, vascular; sign., significance; ns, non-significant; PTC, peritubular capillaries; Art, arterial; Glom, glomerular; TBM, tubular basement membrane; BM, basement membrane. Scale bar = 200µm; *p < 0.05; **p < 0.01; ****p < 0.0001.

**Figure 8:**
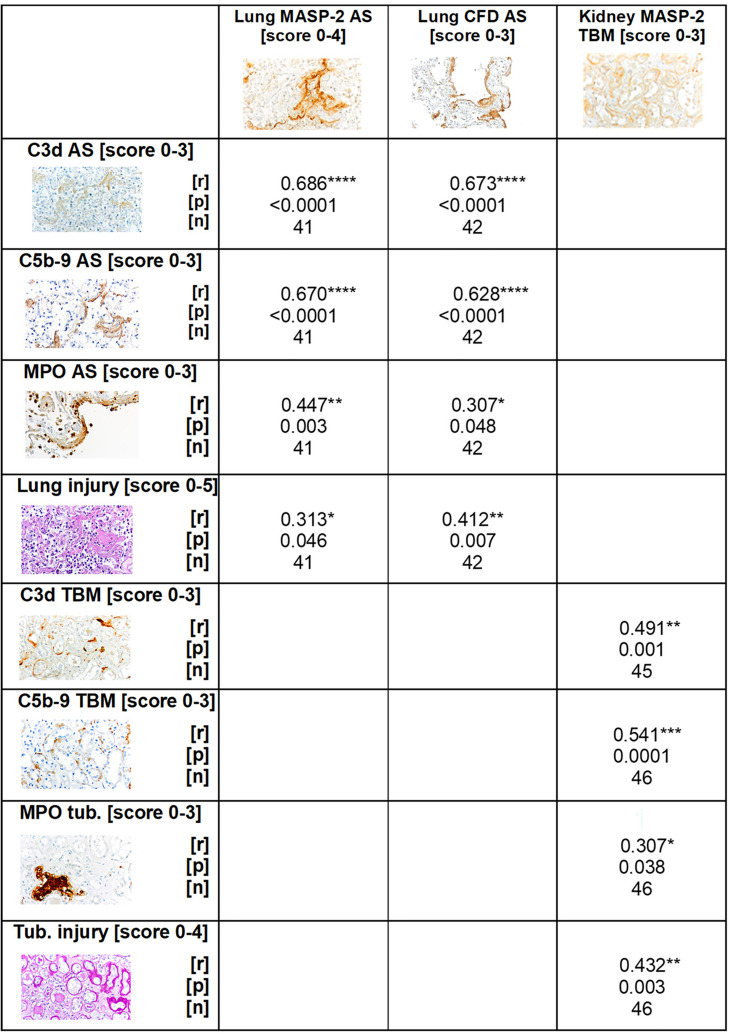
Correlation of MASP-2 and CFD deposition with C3d, C5b-9, MPO-positive cells and tissue injury. Scores for MASP-2 as well as CFD-positive alveolar septa in lungs and MASP-2-positive tubular basement membrane in the kidney correlated with scores of C3d, C5b-9, MPO staining in the same compartment, respectively, and with lung or tubular injury score using Spearman test. AS, alveolar septa; TBM, tubular basement membrane; Tub, tubular lumen; r = correlation coefficient; p = p-value (significance level), n = sample size. *p < 0.05; **p < 0.01; ***p < 0.001, ****p < 0.0001.

To investigate the terminal complement pathway, the deposition of C5b-9 in lung and kidney was analyzed. The sum score for this complement factor, however, was not significantly different between the samples of patients with COVID-19 and the control group (**Figure 7C**). In parallel to the localization of the other complement factors C3d and MASP-2, we detected C5b-9 in the alveolar septa significantly more frequently in the COVID-19 group (**Figures 7D–G**). Surprisingly, however, C5b-9 was found more frequently in the control group in the area of the alveolar inflammatory cells (**Figure 7D**) when compared to COVID-19 patients. In the pulmonary vessel walls, C5b-9 was equally frequently detectable in both groups (**Figures 7A, B, D**). In contrast, C5b-9 was barely detectable in the lumina of the alveoli and completely absent in the bronchioli (**Figure 7D**). Similar to the lungs, no significant difference could be found in the overview pictures (**Figure 7H, I**) between the study groups and with regard to the sum score of C5b-9 in the kidneys (**Figure 7J**). Nevertheless, increased deposition in the peritubular capillaries (PTC) and tubular basement membranes (TBM) was recorded in the samples of COVID-19 patients, similar to MASP-2 and C3d (**Figures 7K–Q**). Since immunohistochemical staining of complement factors requires different antigen retrieval techniques, double staining for co-localization studies was not successful. However, correlation analyses for MASP-2 and CFD with C3d and C5b-9 as well as analysis of the same areas confirm that these four complement factors are deposited in the same way in corresponding compartments in lungs (**Figure 8**), indicating complement activation *via* the lectin and alternative pathways. Co-localization of MASP-2, C3d and C5b-9 in the lung was confirmed by staining of serial sections (**Figure 8**, **Supplementary Figure 2**). However, in kidneys only MASP-2 correlated with C3d and C5b-9, indicating that COVID-19 mediated complement activation is here restricted to the lectin pathway (**Figure 8**).

### Myeloperoxidase (MPO) Positive Neutrophils Were Increased in Lungs and Kidneys From Patients With COVID-19

**Figure 9:**
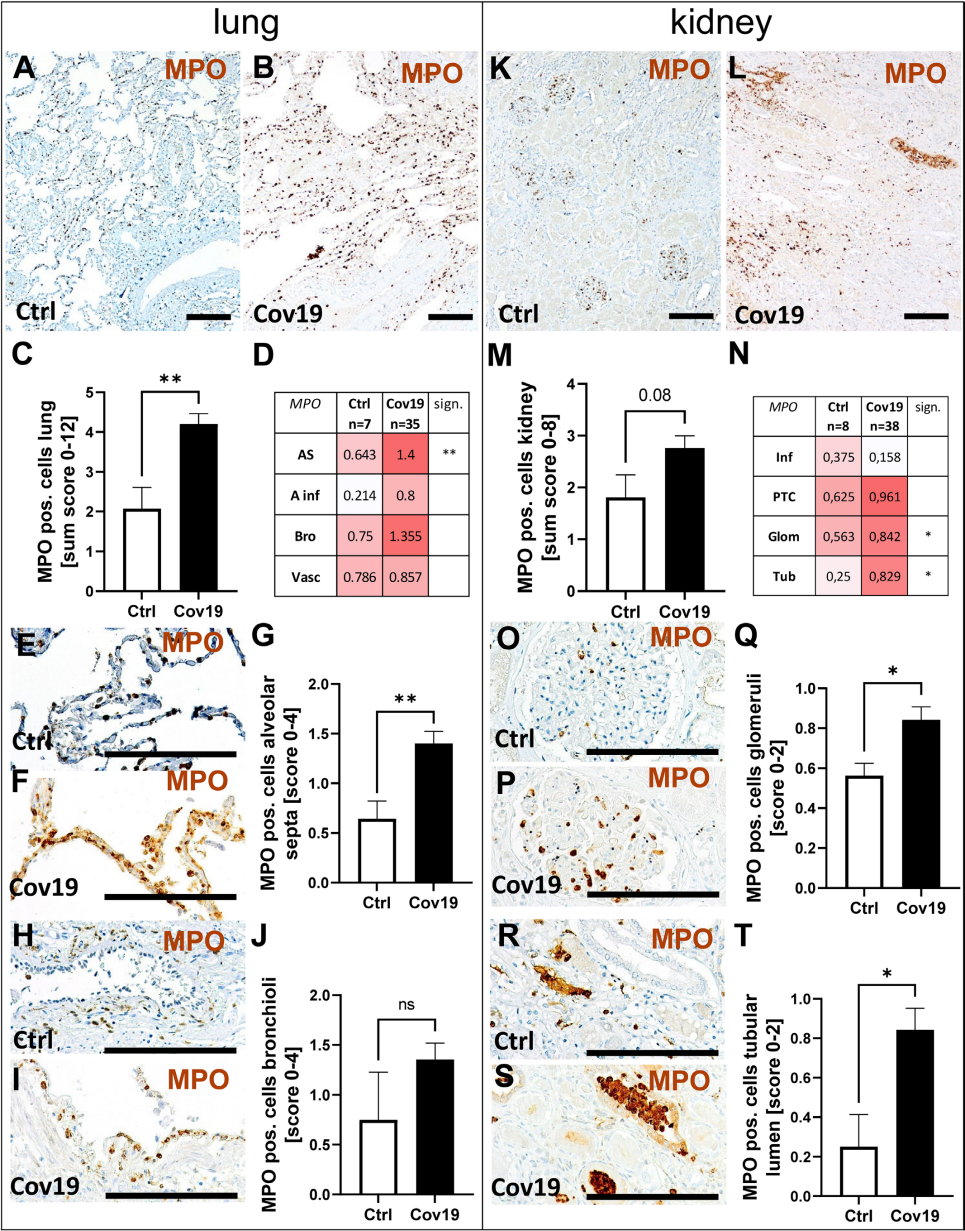
Detection of myeloperoxidase (MPO)-positive cells in lung and kidney specimens of COVID-19 patients. Overview of immunohistochemical staining for MPO staining in lungs of a control patient **(A)** and a patient with COVID-19 **(B)**. Results of semi-quantitative scoring in multiple compartments for MPO-positive cells in the lung summarized in a sum score **(C)** and overview of the mean values of compartment-specific analyses given in a heat map **(D)**. Representative MPO staining in alveolar septa of a control patient **(E)** and a COVID-19 patient **(F)** and statistical comparison of both groups **(G)**. MPO staining in bronchioli in a control patient **(H)** and a patient with COVID-19 **(I, J)**. Overview of MPO staining in kidneys of a control patient **(K)** and a patient with COVID-19 **(L)**. Results of semi-quantitative scoring in multiple compartments for MPO-positive cells in the kidney summarized in a sum score **(M)** and overview of the mean values of compartment-specific analyses given in a heat map **(N)**. Representative MPO staining in glomeruli of a control patient **(O)** and a COVID-19 patient **(P)** and statistical comparison of both groups **(Q)**. MPO positive cells in the lumen of tubuli in a control patient **(R)** and a patient with COVID-19 (**S**, arrows) and statistical comparison of both groups **(T)**. AS, alveolar septa; A inf, alveolar infiltrate; Bro, bronchioles; Vasc, vascular; sign., significance; ns, non-significant; Inf, infiltrate; PTC, peritubular capillaries; Glom, glomerular; Tub, tubular lumen; Scale bar = 200µm; *p < 0.05; **p < 0.01.

Since neutrophils express the C5a receptor 1 on their surface in high density, we evaluated the occurrence of myeloperoxidase (MPO) positive neutrophils as potential C5a-stimulated inflammatory cells. MPO-positive neutrophils were found in large numbers in both groups (**Figures 9A, B**), but were twice as frequent in the sum score in the COVID-19 group compared to controls (**Figure 9C**). In the compartment-specific analysis of neutrophils, infiltration was significantly increased in the alveolar septa (**Figures 9D–G**). Although the number of neutrophils in the alveolar infiltrates and bronchioli of COVID-19 patients was also more than two times higher on average than in the control group, the significance level was not reached due to the large variance observed (**Figures 9D, H–J**). Comparable amounts of MPO-positive cells were found in the large vessels of both groups (**Figure 9D**, Vasc). In the kidney, the infiltration with neutrophils was lower compared to the lung (**Figures 9K, L**). The comparison of both groups in the sum score only showed a tendency towards increased numbers in the COVID-19 group (**Figure 9M**). In contrast, the number of neutrophils in the glomeruli and in the lumina of the tubules was significantly increased in the COVID-19 group (**Figures 9N–T**). Correlation analysis with MASP-2 also showed a significant correlation in both alveolar septa and tubules with the number of MPO-positive cells (**Figure 8**),suggesting that these cells may be locally activated by the complement system. In addition, alveolar MASP-2 and CFD correlated with lung injury and MASP-2 deposition in TBM correlated with tubular injury (**Figure 8**).

In summary, strong deposition of complement factors in lung and kidney samples of patients who died of COVID-19 underlined the potential importance of complement in COVID-19-mediated pathology.

## Discussion

This study to date investigates the largest series of lung and kidney specimens for local deposition of complement components in patients who died of severe COVID-19. The lung is considered the primary site of damage in COVID-19 disease, but there are numerous reports that damage also occurs in other organs (5). In particular, acute tubular injury has been described in the kidney after severe COVID-19 and increase of proteinuria was associated with poor prognosis and increased mortality (6, 7). Here, we therefore investigated local complement over-activation as a common pathomechanism in COVID-19-mediated injury in lung and kidney. We also tried to differentiate the complement pathway operational by which complement was activated by analyzing C1q for the classical and MASP-2 for the lectin pathway and CFD for the alternative pathway. The lectin pathway was activated in both lungs and kidneys of patients with severe COVID-19 in our study. MASP-2 was detectable in the lung mainly in the alveolar septa, the hyaline membranes but also in the lumina of the alveoli. Activation of the lectin pathway was supported by staining for C4d in the same localization as described for MASP-2 (**Supplementary Figure 3**). MASP-2 was strongly expressed in the liver (**Supplementary Figure 1**), while renal and lung MASP-2 expression in cellular components was very low or lacking, as supported by our recent study investigating complement in renal transplants (29) and RNA expression data provided in the web database “Human Protein Atlas” (30). This indicates that extrahepatic expression of MASP-2 is not required for lectin pathway activation. In contrast, other complement factors like C1 and C3 were locally expressed by renal and inflammatory cells (29) and its expression might be important in pathogenesis of complement-mediated injury. Complement activation *via* the lectin pathway was confirmed in an earlier study by Magro et al. investigating local complement activation in small sample numbers of lungs and skin from patients with COVID-19, showing MASP-2 and C4d deposition in intraalveolar septa (23). In addition, C4d staining co-localized with detection of SARS-CoV-2 spike glycoprotein (23), suggesting direct complement activation by SARS-CoV-2 virus in the lung. In experiments *in vitro*, SARS-CoV-2 spike and nucleocapsid protein bind to the recognition components of the lectin pathway namely mannose-binding lectin (MBL), ficolin 2, and collectin-11, whereas C1q does not (17). MASP-2 can then activate protein C4 after binding of these SARS-CoV-2 protein/recognition molecule complexes but also by direct interaction with the nucleocapsid protein (17). Expression of MBL and ficolin-3, both recognition molecules of the lectin pathway, were shown to be upregulated in lungs of patients with COVID-19 (31). While the complement system contributes to a reduction of the viral load in the early phase of the disease by opsonizing the viruses, the complement system can become over-activated during the course of the disease, which then exerts its harmful effects by unleashing a cytokine storm, by direct cell damage *via* the membrane attack complex or by interacting with the coagulation cascade (32). The interaction with the coagulation cascade can promote thrombus formation which is an important feature of COVID-19 pathology, which we mainly observed in the lungs but also in the kidneys of patients with COVID-19, although at much lower rates. The coagulation cascade and the complement cascade are closely linked (33) and the cross talk between the complement system and the coagulation cascade in COVID-19 patients may lead to an enhanced rate of coagulopathies (34). For example, MASP-2 can cleave prothrombin to thrombin (35) and factor XIII and fibrinogen is cleaved by MASP-1 but with significantly lower turnover than observed for the enzymes of the coagulation cascade (36). In addition to activation *via* the lectin pathway in lungs of patients with COVID-19, we established complement activation *via* the alternative pathway by detecting CFD. This is in line with reports from others who have also detected CFD in lungs from patients severely affected by COVID-19 (22, 25). However, in our study COVID-19-induced activation of the alternative pathway was restricted to the lungs, while CFD deposition was comparable in kidneys of both investigated groups. In contrast to our results, these two studies described complement activation *via* the classical pathway in addition (22, 25) and detected activation of the alternative pathway in both lungs and kidneys (22). It is possible that the detection of pathway-specific complement factors is significantly dependent on the antibodies used and the condition and fixation of the tissues. Since antigens are not masked in frozen tissue, they are easier to detect, but non-specific staining also frequently occurs. With C1q established for diagnostic purposes, we would have expected stronger staining for C1q if the classical pathway was to play an important role in COVID-19-mediated damage. Thrombus formation, as a hallmark in COVID-19 pathology, is induced by neutrophil extracellular traps (NETs) formed by activated neutrophils (37). In our study numbers of neutrophils were increased in lung and kidney and correlated with deposition of MASP-2 in both organs. Neutrophils activated by complement anaphylatoxins C5a induce expression of tissue factor, NET formation and thrombosis and could be inhibited by treatment with a C5aR1 inhibitor (38). C3a and C5a cleavage products are formed after activation of all complement pathways, bind to its receptors on inflammatory cells, e.g. neutrophils and macrophages and have a key role in recruiting and activating these cells (39). Serum levels of C5a, indicating complement activity, increased with severity in patients with COVID-19 and hereby inducing expression of cytokines that can elicit a cytokine storm (39). In our study we could confirm positive correlation of complement activation with severity of COVID-19 on organ level since lung injury scores as well as tubular injury scores were associated with increased activation of MASP-2. Blood analyses in patients with COVID-19 suggest that the outcome of the disease depends on the activated complement pathway. Accordingly, a group of patients in whom the alternative and lectin pathways were activated showed more complications and higher mortality than another group in which mainly activity of the classical pathway was detected (40). Complement activation in the kidney was lower compared to the lung and restricted to the lectin pathway. This difference may also be due to the fact that in the lung direct complement activation by the SARS-CoV-2 virus occurs because of a higher virus load. In contrast, in kidneys virus was not (11, 13) or only sporadically (7, 9) detected. Similar findings were made using our autopsy material of patients with severe COVID-19 showing no SARS-CoV-2 spike protein detection in any of the kidneys and in the lungs in only 22% of the cases. This unexpected low virus detection rate might be explained by the different disease durations of the individual patients or by the relatively poor preservation of the autopsy tissue. In the kidney we suggest that complement activation occurs indirectly as a consequence of tissue damage and cell death. Due to the relatively poor preservation of the autopsy tissue, an assessment of acute ischemic damage is not possible, so that we can only speculate whether complement activation is causative of or results secondary from renal injury in severe COVID-19 disease. Complement activation also occurs after hypoxic injury in the kidney as shown for ischemia/reperfusion (41, 42). In earlier studies, we investigated early post transplant biopsies from patients with delayed graft function, showing increased complement activation compared to zero-time biopsies *via* different activation pathways (43). However, it is questionable, if ischemia/reperfusion after transplantation is comparable to COVID-19-induced injury. However, direct complement activation by the virus at an earlier time point before death of the patients cannot be excluded. Our own and data of others underline that complement activation might be an important pathomechanism of lung but also kidney damage in COVID-19 opening the possibility of treatment with complement inhibitors. Currently, only anecdotal reports of the successful treatment of seriously ill patients with COVID-19 employing the MASP-2 inhibitor Narsoplimab (44), the C5 inhibitor Eculizumab (45–47) or the C3 inhibitor AMY-101 (48) have been published. However, more clinical studies using complement inhibitors in patients with COVID-19 evaluating more patients will show the efficiency of this treatment.

## Limitation of the study

Albeit we excluded material with major autolytic changes our study is limited by the quality of the autopsy material, so that some analyses, such as the investigation of capillarization, were not possible. Furthermore, the patients studied had a variety of comorbidities, such as chronic kidney disease, which could possibly also have influenced complement activation, at least in the kidney. Detection of MASP-2 is limited by the fact that the used antibody also recognizes the MASP2 splice variant MAp19, lacking the catalytic domain. Therefore we cannot distinguish between complement activating MASP-2 and MAp19.

In conclusion, in lungs we observed marked complement activation *via* the lectin and alternative pathway in patients who died on COVID-19, while in kidney COVID-19-induced complement activation was restricted to the lectin pathway. Complement deposition was located primarily in capillaries and hyaline membranes in the lung and in peritubular capillaries and on tubular basement membranes in the kidney of COVID-19 patients. Therefore, we speculate that complement is involved in vascular but also epithelial cell damage in both organs of patients with COVID-19. To prevent deregulated complement activation and subsequent collateral tissue injury specific complement inhibition might thus be a promising treatment option.

## Data Availability Statement

The original contributions presented in the study are included in the article/**Supplementary Material**. Further inquiries can be directed to the corresponding author.

## Ethics Statement

Analysis was approved by the local Ethics committees (Ethics committee of the Friedrich-Alexander University (FAU) Erlangen-Nürnberg, reference number 4415 and internal review board of the Medical Center-Augsburg (BKF No. 2020–18) and the ethics committee of the University of Munich (Project number 20–426, COVID-19 registry of the University hospital Augsburg). Written informed consent for participation was not required for this study in accordance with the national legislation and the institutional requirements.

## Author Contributions

JN analyzed data and wrote the manuscript. TR carried out immunohistochemistry staining. CE collected tissue samples and clinical data and revised the manuscript. CD, MB-H, KA, BM, and AH conceived and designed the study, analyzed data and wrote the manuscript. All authors contributed to the article and approved the submitted version.

## Funding

This study was funded by the Deutsche Forschungsgemeinschaft (DFG, German Research Foundation), project number 387509280, SFB 1350 and supportet by the DEFEAT Pandemics autopsy platform of the Federal Ministry of Education and Research (BMBF).

## Conflict of Interest

The authors declare that the research was conducted in the absence of any commercial or financial relationships that could be construed as a potential conflict of interest.

## Publisher’s Note

All claims expressed in this article are solely those of the authors and do not necessarily represent those of their affiliated organizations, or those of the publisher, the editors and the reviewers. Any product that may be evaluated in this article, or claim that may be made by its manufacturer, is not guaranteed or endorsed by the publisher.
