# Corrigendum: Complement activation via the lectin and alternative pathway in patients with severe COVID-19

**DOI:** 10.3389/fimmu.2023.1294153

**Published:** 2023-09-21

**Authors:** Janina Niederreiter, Christine Eck, Tajana Ries, Arndt Hartmann, Bruno Märkl, Maike Buettner-Herold, Kerstin Amann, Christoph Daniel

**Affiliations:** ^1^Department of Nephropathology, University Hospital Erlangen, Friedrich-Alexander-University (FAU) Erlangen-Nürnberg, Erlangen, Germany; ^2^Institute of Pathology, University Hospital Erlangen, Friedrich-Alexander-University (FAU) Erlangen-Nürnberg, Erlangen, Germany; ^3^General Pathology and Molecular Diagnostics, Medical Faculty Augsburg, University Augsburg, Augsburg, Germany

**Keywords:** complement deposition, COVID-19, kidney, lung, autopsy, lectin pathway

In the published article, information in the **Acknowledgments** was mistakenly not included in the publication. A correction has been made to **Acknowledgments**:

