# Supplementary figures and images for: Complement Activation via the Lectin and Alternative Pathway in Patients With Severe COVID-19
Source: Front Immunol. 2022 Feb 2;13:835156. doi: 10.3389/fimmu.2022.835156 (PMC8884149; doi:10.3389/fimmu.2022.835156)

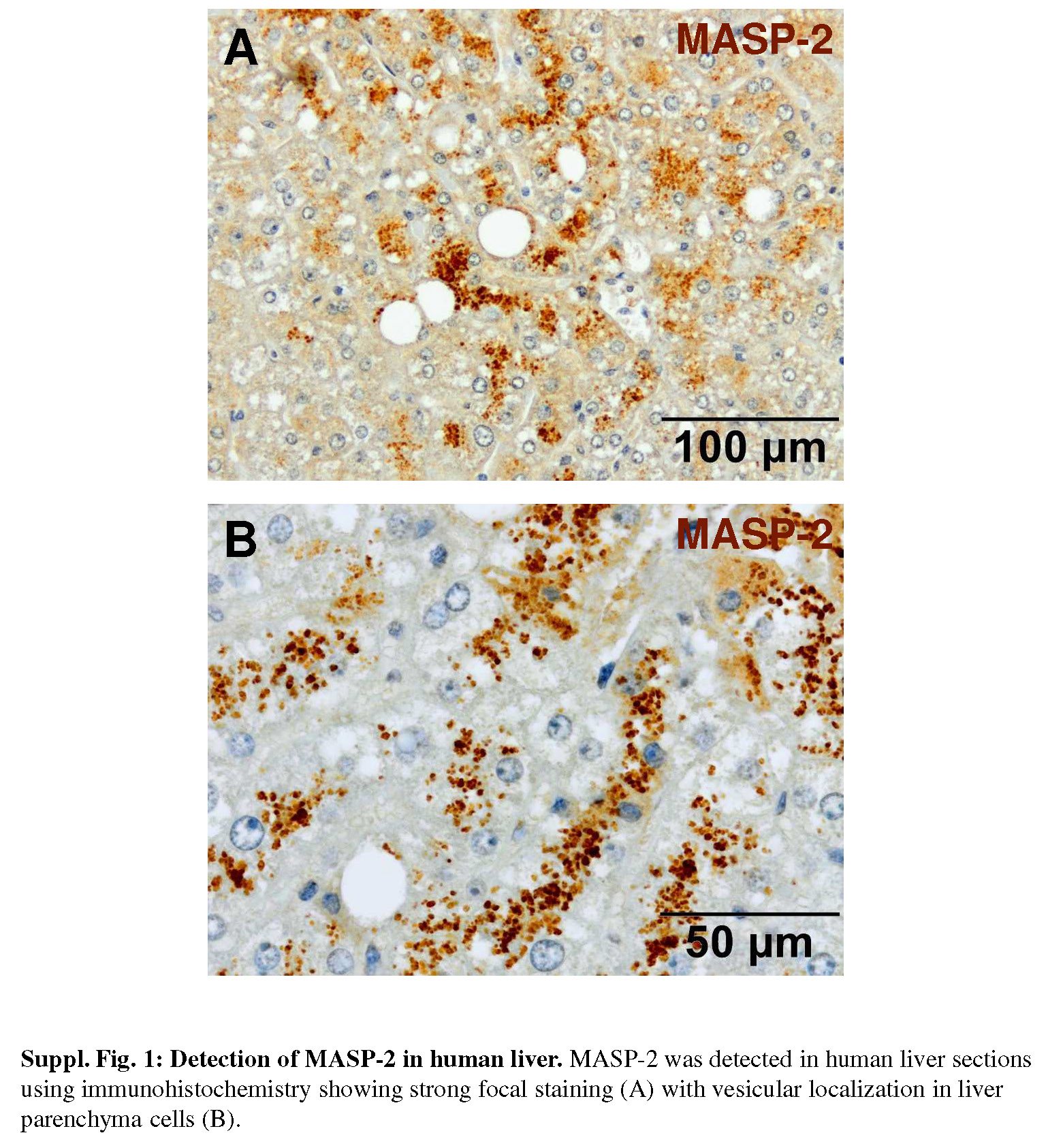

Supplement: Supplementary file 1 [file Image_1.jpg]

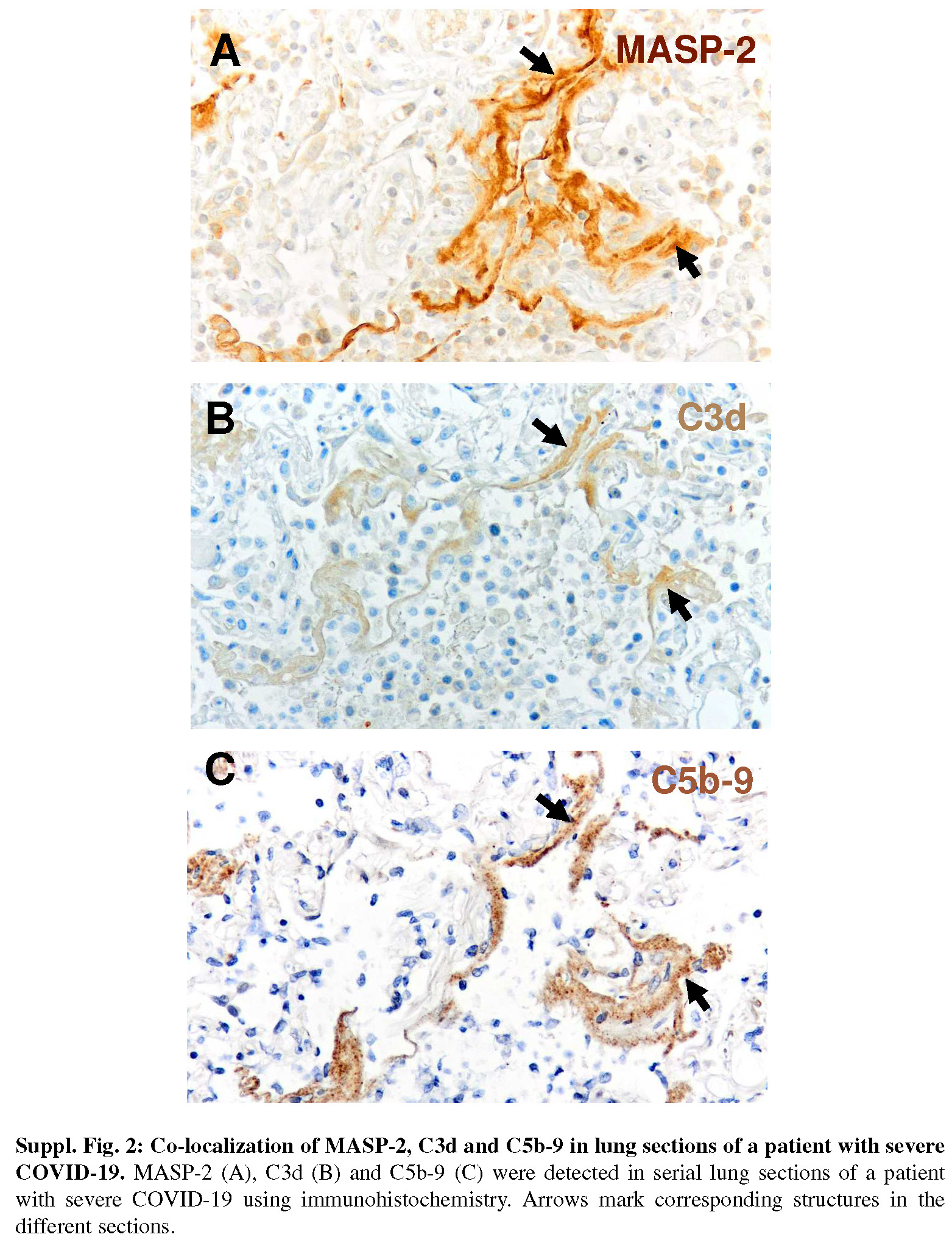

Supplement: Supplementary file 2 [file Image_2.jpg]

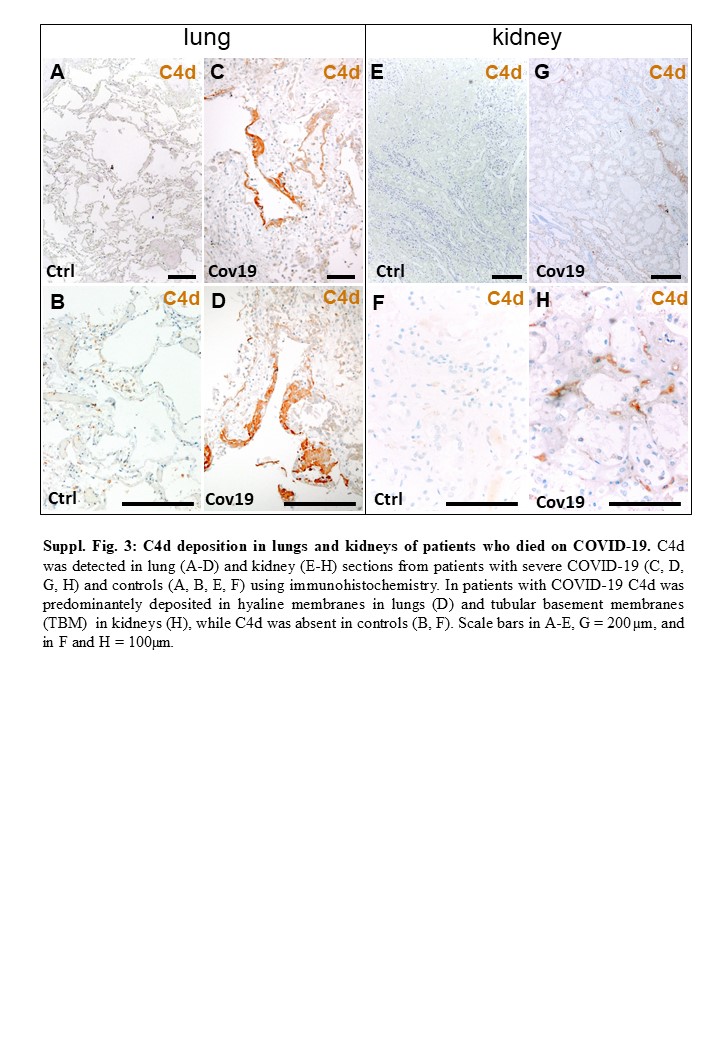

Supplement: Supplementary file 3 [file Image_3.jpeg]
